# Association between a single nucleotide polymorphism of the IL23R gene and tuberculosis in a Chinese Han population: a case‒control study

**DOI:** 10.1186/s12890-023-02546-w

**Published:** 2023-07-18

**Authors:** Juan Zhang, Ming-Gui Wang, Xi Xiang, Jian-Qing He

**Affiliations:** 1 Department of Respiratory and Critical Care Medicine, West China Hospital of Sichuan University, No. 37, Guo Xue Alley, Chengdu, 610041 Sichuan Province People’s Republic of China; 2 Intensive Care Unit, Deyang People’s Hospital, No 173, North Taishan Road, Deyang, 618000 Sichuan Province People’s Republic of China; 3 West China School of Nursing, West China Hospital of Sichuan University, No. 37, Guo Xue Alley, Chengdu, 610041 China

**Keywords:** IL23R gene, Single nucleotide polymorphism, Tuberculosis, Severity

## Abstract

**Background:**

Severe tuberculosis constitutes a significant menace to human safety and well-being, with a considerable mortality rate. The severity of tuberculosis can be impacted by genetic variations in host genes, particularly single nucleotide polymorphisms (SNPs).

**Methods:**

A case‒control study was undertaken, encompassing a cohort of 1137 tuberculosis patients (558 with severe tuberculosis and 579 with mild tuberculosis), alongside 581 healthy controls within the age range of fifteen to forty-five years. Whole blood DNA was extracted from all participants, and three tag polymorphisms (rs1884444, rs7518660, rs7539625) of the *IL23R* gene were selectively identified and genotyped.

**Results:**

No significant correlation was observed between the *IL23R* gene polymorphisms (rs1884444, rs7518660, and rs7539625) and tuberculosis. Upon comparing the tuberculosis group with the healthy control group, the mild tuberculosis group with the healthy control group, and the severe tuberculosis group with the healthy control group, the obtained P-values were> 0.05. However, in the comparison between severe tuberculosis and mild tuberculosis, the presence of rs1884444 G alleles exhibited a significantly increased risk of severe tuberculosis after adjusting for age and sex (OR^a^: 1.199, 95% CI: 1.009–1.424; P^a^=0.039, respectively). In subgroup analysis, after accounting for confounding factors, including age and sex, rs1884444 G alleles continued to demonstrate a significantly heightened risk of severe tuberculosis. Nonetheless, the comparison between the multisystemic tuberculosis group and the mild tuberculosis group was no significant difference. Notably, rs1884444 of the *IL23R* gene exhibited a noteworthy association with the risk of severe tuberculosis in the comparison between severe tuberculosis and mild tuberculosis before and after adjusting for age and sex (OR^a^: 1.301, 95% CI: 1.030–1.643; P^a^=0.027, respectively). Furthermore, the presence of the rs1884444 G allele exhibited a significantly increased risk of severe tuberculosis after adjusting for age and sex in the comparison between tuberculous meningitis and mild tuberculosis (OR^a^: 1.646, 95% CI: 1.100-2.461; P^a^=0.015, respectively).

**Conclusions:**

The present study suggests that there is no significant association between *IL23R* gene polymorphism and tuberculosis susceptibility in the Chinese Han population. However, it does indicate a potential link between *IL23R* polymorphism and an increased risk of developing severe tuberculosis.

**Supplementary Information:**

The online version contains supplementary material available at 10.1186/s12890-023-02546-w.

Tuberculosis (TB) represents a significant global public health dilemma and stands as one of the foremost causes of mortality worldwide. In 2021, the global count of newly reported TB cases amounted to 10.6 million, with China contributing 780,000 cases, ranking third among the 30 nations facing a substantial TB burden, falling behind only Indonesia and India [1]. Global TB fatalities in 2021 reached 1.6 million, yielding a TB fatality rate of 15%, surpassing the figures for both 2020 (1.5 million) and 2019 (1.4 million), effectively returning to levels last observed in 2017 [1]. Despite the rapid advancements in TB diagnostic and therapeutic technology, there are still exist patients who develop severe TB due to various factors, resulting in imminent respiratory failure and dysfunction of extrapulmonary organs, thereby gravely endangering the lives of afflicted individuals [2]. Consequently, combating severe TB assumes paramount significance as a public health initiative, aligning with the objective of the World Health Organization’s “Stop TB” campaign, slated for achievement by 2035.

SNPs are widely acknowledged as the primary wellspring of human genetic variation. Recent years have witnessed the emergence of compelling evidence, derived from comprehensive genome-wide linkage analysis studies, candidate gene association studies, and genome-wide association analyses, substantiating the notion that host gene polymorphisms can exert a profound impact on the onset, progression, and clinical outcome of TB [3]. Host gene SNPs have the potential to modulate an individual’s susceptibility and the severity of TB manifestations. At present, severe TB confronts several challenges. Firstly, the lack of unified definitions, standards, and guidelines results in divergent interpretations among researchers. Secondly, the mortality rate associated with severe TB remains alarmingly high. Thirdly, clinical practitioners often fail to accord adequate attention to severe TB cases. Fourthly, the intricate pathogenesis of severe TB involves numerous genetic mechanisms that remain elusive. Consequently, the objective of our study centers around delving deeper into the influence exerted by host gene polymorphisms on TB susceptibility and severity within the Chinese Han population.

## Methods

### Ethics statement

The present investigation received the ethical endorsement of the ethics committee of West China Hospital of Sichuan University [Approval No.: 932 (2019)]. All methodologies were conducted in strict adherence to pertinent guidelines and regulations. The current research was conducted in accordance with the guiding principles of the Declaration of Helsinki. The participants were adequately informed about the study’s purpose and implementation strategy, after which they provided their informed consent by signing consent forms. In cases where the participant was under the age of eighteen, the consent forms were signed by the legal guardian. Similarly, if a participant lacked the capacity to provide consent due to illness, the consent forms were signed by their representative.

### Study design

The study followed a case‒control design and comprised a total of 1137 TB patients at West China Hospital of Sichuan University between January 2013 and December 2020. Among them, 558 patients were afflicted with severe TB, while 579 patients exhibited mild TB symptoms. Healthy controls were recruited from the People’s Hospital of DeYang City in Sichuan Province for annual physical examination. All participants belonged to the Han Chinese ethnic group.

### Study population

The criteria for patient inclusion were as follows: 1) signed written consent; 2) ≥ 15 years old and ≤ 45 years old; and 3). Tuberculosis group included both clinical diagnosis and bacteriologically confirmed patients. The clinical diagnosis of TB is defined as the high suspicion of TB based on the patient’s symptoms, signs, laboratory examination, imaging examination, etc., excluding tumor, non-tuberculosis mycobacterium and fungal infection. These patients improved after anti-tuberculosis therapy. Bacteriologically confirmed is defined as confirmation by TB DNA, gene Xpert, TB culture or histopathology [4, 5]. Patients outside the age range of 15 to 45 were excluded from the study. Additionally, patients with hepatitis B, cirrhosis, tumors, HIV, immune system disorders, pneumoconiosis, renal insufficiency, or who had undergone organ transplantation was also excluded. If the participants are related, they will be excluded. Currently, there is a paucity of relevant definitions, standards, and guidelines pertaining to severe tuberculosis. In this study, we have operationalized severe tuberculosis as encompassing any of the following clinical presentations, as identified in pertinent literature [2, 6–9]: (1) pulmonary lesions affecting more than two-thirds of the lung as determined by imaging, (2) the presence of a cavity exceeding 4 cm in size, (3) hematogenous disseminated pulmonary tuberculosis, (4) the diagnosis of tuberculous meningitis, and (5) involvement of multiple systems (two or more systems). Conversely, individuals failing to meet these specified criteria are classified as having mild TB.

### Genotyping

We utilized the NCBI 1000 Genomes Project (https://www.ncbi.nlm.nih.gov/) to select haplotype-tagged SNPs from the CHB (Han Chinese in Beijing) population. Specifically, we focused on a specific region within the IL23R gene located on chromosome 1p31.3. The selection of tagged SNPs was carried out with a linkage disequilibrium r2 cutoff of 0.8 and a minor allele frequency (MAF) of ≥ 5%. Three tag SNPs, namely rs1884444, rs7518660, and rs7539625, were chosen within the IL23R region. DNA was extracted by TianGen kit (Tiangen Biotech Beijing, Co., Ltd, China) and then stored in a -80 °C freezer for further study. SNPscanTM was used for multiple SNP typing. SNPs genotyping were performed utilizing SNPscanTM Kit(Cat#: G0104K, Genesky Inc. Shanghai, China).The basic principle of this technique is to recognize alleles at SNP sites with high specificity of ligase binding reactions [10].

### Statistical analyses

Data analysis was performed using SPSS 27.0 statistical software (SPSS, Chicago, IL, USA). The comparison of baseline data characteristics between the TB group and control group employed Student’s t-test for continuous variables, while chi-square tests and Fisher’s exact probability were utilized for categorical variables. To assess the deviation from Hardy-Weinberg equilibrium, a χ2 test was conducted to compare observed and expected genotype frequencies in the tuberculosis and control groups. A significance level of P < 0.05 was considered statistically significant. Furthermore, P values, odds ratios (OR) and 95% confidence intervals (95% CI) of the association between SNPs and TB susceptibility or severity were calculated using binary logistic regression. This analysis was performed for allele, genotype, and three genetic models (dominant, recessive, and additive models), with adjustments made for age and sex. In addition to comparing the TB group with the healthy control group, a subgroup analysis was conducted. To evaluate the linkage disequilibrium (LD) and perform haplotype analysis between SNPs, the SHEsis online software platform was utilized [11].

## Results

### Characteristics of TB patients and controls

Our study encompassed a case‒control design, enrolling a total of 1137 TB patients and 581 healthy controls. The TB group had a mean age of 27.92 ± 8.254 years, while the healthy control group had a mean age of 27.97 ± 6.093 years. In the TB group, there were 606 males (53.3%) and 531 females (46.7%), whereas the healthy control group consisted of 302 males (52%) and 279 females (48%). No significant differences were observed in terms of age (P = 0.889) or sex (P = 0.604) between the two groups. Based on our previous definition, TB patients were categorized as severe TB (558 cases) and mild TB (579 cases). Among the 558 cases of severe TB, there were 219 cases of severe pulmonary tuberculosis (PTB), 286 cases of multisystem TB, and 53 cases of tuberculous meningitis (Supplementary Table 1). The sample qualification rate was 98.47%, and the success rate ranged from 99.23 to 100%. Additionally, no deviations from the Hardy-Weinberg equilibrium were observed in the control group (P > 0.05).

### Association between ***IL23R*** SNPs and TB susceptibility

Table 1 presents the association between *IL23R* SNPs and TB susceptibility. Our findings revealed no statistically significant differences in allele frequencies or genotype distribution frequencies among the two groups. Moreover, the genetic models analyzed including dominant, recessive, and additive models, no statistically significant differences were identified. We further conducted comparisons between the mild tuberculosis group and the healthy control group, as well as the severe tuberculosis group and the healthy control group. However, no statistically significant differences in allele frequencies, genotypic distribution frequencies, or other genetic models analyzed were observed in these comparisons either. Therefore, we did not observe any association between the selected *IL23R* SNPs and susceptibility to TB. Supplementary Table 2 displays the gene distribution frequencies of the mild TB group versus the healthy control group and the severe TB group versus the healthy control group.

**Table 1:** Analysis of IL23R gene SNPs in association with susceptibility to TB

SNPs	Genetic models	Allele/ Genotype	TB group N(%)	Healthy control group N(%)	OR(95%CI)	P	OR^a^(95% CI)	P^a^	Mild TB group vs. Healthy control group				Severe TB group vs. Healthy control group			
									OR(95%CI)	P	OR^a^(95% CI)	P^a^	OR(95%CI)	P	OR^a^(95% CI)	P^a^
rs1884444(T>G)		T	1433(63.0)	731(62.9)	1	Reference	1	Reference	1	Reference	1	Reference	1	Reference	1	Reference
		G	841(37.0)	431(37.1)	0.995(0.860–1.152)	0.951	0.995(0.859–1.152)	0.994	0.909(0.767–1.077)	0.267	0.909(0.767–1.078)	0.273	1.092(0.922–1.293)	0.310	1.101(0.929–1.305)	0.268
		TT	461(40.5)	234(40.03)	1	Reference	1	Reference	1	Reference	1	Reference	1	Reference	1	Reference
		TG	511(44.9)	263(45.3)	0.986(0.794–1.225)	0.900	0.986(0.794–1.224)	0.895	0.904(0.705–1.158)	0.425	0.917(0.715–1.176)	0.494	1.084(0.841–1.396)	0.534	1.091(0.846–1.407)	0.502
		GG	165(14.5)	84(14.5)	0.997(0.734–1.354)	0.985	0.996(0.733–1.353)	0.979	0.836(0.584–1.196)	0.327	0.828(0.578–1.187)	0.305	1.188(0.837–1.687)	0.335	1.209(0.850–1.720)	0.291
	Additive	2GG + TGvsTT			0.996(0.862–1.150)	0.951	0.995(0.861–1.149)	0.944	0.911(0.711–1.077)	0.277	0.912(0.771–1.078)	0.280	1.089(0.922–1.286)	0.318	1.098(0.928–1.298)	0.275
	Dominant	TG + GGvsTT			0.989(0.807–1.212)	0.914	0.988(0.806–1.211)	0.908	0.887(0.703–1.121)	0.316	0.895(0.708–1.131)	0.354	1.109(0.874–1.407)	0.395	1.119(0.881–1.422)	0.356
	Recessive	GGvsTT + TG			0.995(0.927–1.069)	0.898	0.995(0.927–1.069)	0.893	0.972(0.895–1.054)	0.490	0.977(0.899–1.060)	0.571	1.021(0.939–1.110)	0.625	1.023(0.941–1.112)	0.600
Rs7518660(G>A)		G	1684(74.1)	859(73.9)	1	Reference	1	Reference	1	Reference	1	Reference	1	Reference	1	Reference
		A	590(25.9)	303(26.1)	0.993(0.845–1.167)	0.934	0.991(0.844–1.164)	0.912	0.965(0.801–1.162)	0.705	0.963(0.799–1.160)	0.689	1.023(0.849–1.233)	0.808	1.025(0.850–1.237)	0.793
		GG	624(54.9)	318(54.7)	1	Reference	1	Reference	1	Reference	1	Reference	1	Reference	1	Reference
		GA	436(38.3)	223(38.4)	1.019(0.680–1.529)	0.926	0.993(0.805–1.226)	0.951	0.974(0.765–1.241)	0.833	0.986(0.773–1.257)	0.908	1.020(0.799–1.303)	0.874	1.022(0.800-1.307)	0.862
		AA	77(6.8)	40(6.9)	1.016(0.671–1.538)	0.941	0.978(0.652–1.466)	0.913	0.914(0.569–1.466)	0.708	0.886(0.551–1.425)	0.618	1.053(0.661–1.678)	0.828	1.057(0.662–1.687)	0.817
	Additive	2AA + GAvsGG			0.993(0.846–1.167)	0.934	0.991(0.844–1.164)	0.912	0.965(0.801–1.162)	0.705	0.963(0.799–1.160)	0.689	1.023(0.850–1.232)	0.809	1.025(0.851–1.236)	0.794
	Dominant	GA + AAvsGG			0.994(0.813–1.215)	0.953	0.991(0.811–1.212)	0.930	0.965(0.766–1.216)	0.763	0.970(0.769–1.224)	0.799	1.025(0.812–1.294)	0.836	1.027(0.813–1.298)	0.821
	Recessive	AAvsGA + GG			0.999(0.931–1.071)	0.976	0.998(0.930–1.070)	0.954	0.992(0.915–1.075)	0.844	0.996(0.919–1.080)	0.922	1.006(0.928–1.092)	0.881	1.007(0.928–1.093)	0.869
Rs7539625(A>G)		A	1158(50.9)	621(53.4)	1	Reference	1	Reference	1	Reference	1	Reference	1	Reference	1	Reference
		G	1116(49.1)	541(46.6)	1.106(0.960–1.274)	0.162	1.103(0.957–1.271)	0.174	1.086(0.923–1.278)	0.320	1.080(0.917–1.272)	0.356	1.127(0.956–1.329)	0.153	1.134(0.961–1.337)	0.137
		AA	306(26.9)	170 (29.3)	1	Reference	1	Reference	1	Reference	1	Reference	1	Reference	1	Reference
		GA	546(48.0)	281(48.4)	1.079(0.852–1.367)	0.526	1.072(0.846–1.359)	0.563	1.040(0.792–1.366)	0.779	1.035(0.788–1.360)	0.805	1.123(0.851–1.481)	0.412	1.112(0.842–1.469)	0.454
		GG	285(25.1)	130(22.4)	1.218(0.921–1.611)	0.167	1.212(0.916–1.604)	0.178	1.177(0.854–1.622)	0.319	1.164(0.844–1.606)	0.354	1.263(0.912–1.748)	0.159	1.278(0.922–1.771)	0.141
	Additive	2GG + GAvsAA			1.102(0.959–1.267)	0.170	1.100(0.956–1.264)	0.182	1.083(0.923–1.271)	0.330	1.077(0.917–1.264)	0.365	1.124(0.955–1.332)	0.159	1.130(0.960–1.330)	0.143
	Dominant	GG + GAvsAA			1.123(0.900-1.402)	0.304	1.116(0.894–1.394)	0.331	1.083(0.839–1.398)	0.539	1.076(0.833–1.390)	0.575	1.167(0.900-1.514)	0.244	1.164(0.897–1.511)	0.254
	Recessive	GGvsGA + AA			1.010(0.936–1.089)	0.805	1.008(0.934–1.087)	0.846	0.999(0.916–1.090)	0.988	0.999(0.915–1.090)	0.977	1.020(0.934–1.114)	0.654	1.016(0.930–1.110)	0.730

Abbreviation: TB tuberculosis; SNPs single nucleotide polymorphisms; OR odds ratio; CI confidence interval; ^a^, adjusted for age and gender

### Association between ***IL23R*** SNPs and TB severity

Table 2 presents the comparison between 558 cases of severe TB and 579 cases of mild TB in terms of allelic, genotypic frequencies, and genetic model analyzed. We observed a significant association between the minor allele G of rs1884444 and an increased risk of severe TB when compared to allele T (OR: 1.201, 95% CI: 1.013–1.424; P = 0.035). This association remained significant after adjusting for age and sex (OR^a^: 1.199, 95% CI: 1.009–1.424; P^a^ = 0.039). The rs1884444 also exhibited a significant association with an increased risk of severe TB in additive models (OR^a^: 1.194, 95% CI: 1.009–1.412; P^a^: 0.039). The association remained statistically significant after adjusting for age and sex (OR^a^: 1.192, 95% CI: 1.006–1.412; P^a^=0.043). However, no significant differences were observed in other genetic model analyzed, including the dominant and recessive models, between severe and mild TB, with P values >0.05 after adjusting for age and sex.Regarding the distribution frequencies of alleles, genotypes, and genetic models (additive model, dominant model, and recessive model) of rs7518660 and rs7539625, no significant differences were found between severe and mild TB, with P values >0.05 after adjusting for age and sex.

**Table 2:** Analysis of IL23R gene polymorphisms and severity of tuberculosis

SNPs	Genetic models	Allele/ Genotype	Severe TB group N(%)	Mild TB group N(%)	OR^a^(95% CI)	P^a^	Severe PTB group vs. Mild TB group			Multisystem TB group vs. Mild TB group			tubercular meningitis group vs. Mild TB group	
							OR^a^(95% CI)	P^a^		OR^a^(95% CI)	P^a^		OR^a^(95% CI)	P^a^
rs1884444(T>G)		T	679(60.8)	754(65.1)	1	Reference	1	Reference		1	Reference		1	Refer1ence
		G	437(39.2)	404(34.9)	1.199(1.009–1.424)	0.039	1.301(1.030–1.643)	0.027		1.055(0.954–1.168)	0.297		1.646(1.100-2.461)	0.015
		TT	211(37.8)	250(43.2)	1	Reference	1	Reference		1	Reference		1	Reference
		GT	257(46.1)	254(43.9)	1.195(0.927–1.542)	0.170	1.106(0.782–1.564)	0.568		1.172(0.863–1.592)	0.309		1.667(0.860–3.231)	0.130
		GG	90(16.1)	75(13.0)	1.418(0.988–2.033)	0.058	1.738(1.104–2.737)	0.017		1.025(0.646–1.627)	0.917		2.635(1.173–5.922)	0.019
	Additive	2GG + TGvsTT			1.192(1.006–1.412)	0.043	1.276(1.022–1.592)	0.032		1.056(0.856–1.302)	0.612		1.627(1.090–2.427)	0.017
	Dominant	TG + GGvsTT			1.246(0.981–1.583)	0.072	1.249(0.906–1.723)	0.175		1.139(0.852–1.522)	0.381		1.884(1.010–3.511)	0.046
	Recessive	GGvsTT + TG			1.050(0.966–1.142)	0.254	1.013(0.906–1.133)	0.820		1.055(0.954–1.168)	0.297		1.128(0.922–1.381)	0.243
Rs7518660(G>A)		G	820(73.5)	864(74.6)	1	Reference	1	Reference		1	Reference		1	Reference
		A	296(26.5)	294(25.4)	1.071(0.887–1.295)	0.476	1.182(0.922–1.516)	0.187		0.961(0.761–1.214)	0.740		1.291(0.833-2.000)	0.253
		GG	302(54.1)	322(55.6)	1	Reference	1	Reference		1	Reference		1	Reference
		GA	216(38.7)	220(38.0)	1.005(0.824–1.351)	0.672	1.096(0.787–1.527)	0.586		0.960(0.710–1.297)	0.789		1.387(0.768–2.503)	0.278
		AA	40(7.2)	37(6.4)	1.180(0.731–1.905)	0.497	1.576(0.865–2.873)	0.137		0.926(0.503–1.704)	0.805		1.528(0.499–4.680)	0.458
	Additive	2AA + GAvsGG			1.071(0.887–1.294)	0.476	1.182(0.922–1.516)	0.187		0.961(0.760–1.215)	0.739		1.302(0.834–2.032)	0.246
	Dominant	GA + AAvsGG			1.073(0.847–1.358)	0.559	1.162(0.849–1.591)	0.349		0.955(0.716–1.274)	0.753		1.406(0.797–2.480)	0.239
	Recessive	AAvsGA + GG			1.017(0.937–1.104)	0.690	1.027(0.921–1.146)	0.628		0.987(0.893–1.091)	0.794		1.111(0.914–1.350)	0.289
Rs7539625(A>G)		A	563(50.4)	595(51.4)	1	Reference	1	Reference		1	Reference		1	Reference
		G	553(49.6)	563(48.5)	1.050(0.889–1.239)	0.567	1.145(0.917–1.430)	0.231		1.122(0.916–1.373)	0.265		1.206(0.808–1.801)	0.360
		AA	141(25.3)	144(24.9)	1	Reference	1	Reference		1	Reference		1	Reference
		GA	271(48.6)	275(47.5)	0.987(0.739–1.318)	0.927	1.021(0.695-1.500)	0.915		1.025(0.730–1.438)	0.887		2.162(1.001–4.668)	0.050
		GG	146(26.2)	160(27.6)	0.912(0.658–1.263)	0.579	1.288(0.840–1.974)	0.246		0.924(0.619–1.378)	0.697		1.537(0.626–3.774)	0.348
	Additive	2GG + GAvsAA			1.048(0.890–1.233)	0.574	1.137(0.916–1.410)	0.244		0.964(0.790–1.175)	0.716		1.199(0.808–1.778)	0.368
	Dominant	GG + GAvsAA			1.087(0.834–1.417)	0.537	1.112(0.778–1.590)	0.559		0.990(0.721–1.361)	0.953		1.946(0.924–4.096)	0.080
	Recessive	GGvsGA + AA			1.020(0.932–1.116)	0.669	0.981(0.870–1.107)	0.760		1.017(0.912–1.133)	0.767		1.261(1.006–1.582)	0.045

Abbreviation: TB tuberculosis; SNPs single nucleotide polymorphisms; OR odds ratio; CI confidence interval; ^a^, adjusted for age and gender

### Association between ***IL23R*** SNPs and TB severity subgroup analysis

To further investigate the association between *IL23R* polymorphisms and TB severity, subgroup analyses were conducted comparing severe TB versus mild TB, multisystem TB versus mild TB, and tuberculous meningitis versus mild TB. Table 2 presents the results regarding allelic, genotypic frequencies, and genetic models analyzed between severe and mild TB. In the case of severe pulmonary tuberculosis (PTB) versus mild TB, the minor allele G of rs1884444 exhibited a significant association with an increased risk of severe PTB compared to allele T after adjusting for age and sex (OR^a^: 1.301, 95% CI: 1.030–1.643; P^a^=0.027, respectively). The rs1884444 GG genotype also showed a significant association with an increased risk of severe PTB compared to the TT genotype after adjusting for age and sex (OR^a^: 1.738, 95% CI: 1.104–2.737; P^a^ = 0.017). In the genetic model analysis, rs1884444 demonstrated a significant association with an increased risk of severe PTB in additive models (OR^a^: 1.276, 95% CI: 1.022–1.592; P^a^: 0.032, respectively). However, no significant differences were observed in the distribution frequencies of alleles, genotypes, and gene models (additive model, dominant model, and recessive model) of rs7518660 and rs7539625 between severe PTB and mild TB, with P values exceeding 0.05 after adjusting for age and sex.

Regarding multisystem TB versus mild TB, as well as tuberculous meningitis versus mild TB, no significant differences were found in the distribution frequencies of alleles, genotypes, and genentic models (additive model, dominant model, and recessive model) of rs1884444, rs7518660, and rs7539625 after adjusting for age and sex, with P values exceeding 0.05. However, the minor allele G of rs1884444 showed a significant association with an increased risk of severe TB compared to allele T in the tubercular meningitis group versus the mild TB group after adjusting for age and sex (OR^a^: 1.646, 95% CI: 1.100-2.461; P^a^=0.015, respectively). The rs1884444 GG genotype also showed a significant association with an increased risk of tubercular meningitis compared to the TT genotype after adjusting for age and sex (OR^a^: 2.653, 95% CI: 1.173–5.922; P^a^ = 0.019). In the genetic model analysis, rs1884444 displayed a significant association with an increased risk of severe TB in additive models (OR^a^: 1.627, 95% CI: 1.090–2.427; P^a^: 0.017) and dominant models (OR^a^: 1.884, 95% CI: 1.010–3.511; P^a^: 0.046). Additionally, in the rs7539625 gene model analysis, the recessive models showed significance in the comparison between tuberculous meningitis and mild TB (OR^a^: 1.261, 95% CI: 1.006–1.582; P^a^: 0.045). No significant differences were observed in the distribution frequencies of alleles, genotypes, and genetic models (additive model, dominant model and recessive model) of rs7518660. For detailed gene distribution frequencies of the subgroup analyses, please refer to Supplementary Table 2.

### LD and haplotype analysis

Supplementary Fig. 1 presents the LD analysis between the tagSNPs of the *IL23R* gene. The R^2^ between rs7518660 and rs1884444 was 0.141, indicating a moderate level of LD. The R^2^ value between rs1884444 and rs7539625 was 0.403, indicating a relatively stronger level of LD. Finally, the R^2^ between rs7518660 and rs7539625 was 0.314, indicating a moderate level of LD as well. Haplotypes with frequencies below 0.03 were excluded from the analysis. Table 3 presents the results of the haplotype analysis. Six haplotypes were identified, namely GAG, GGA, GGG, TAG, TGA, and TGG. Among these haplotypes, it was found that haplotype TGGs were significantly associated with a reduced risk of severe tuberculosis ^(^OR^a^: 0.715, 95% CI: 0.526–0.973; P^a^=0.032). However, no significant associations were observed for the other three haplotypes (GAG, GGA, GGG, TAG and TGA).

**Table 3:** Haplotype analysis of IL23R gene SNPs associated with the severity of TB

Haplotypes	Severe TB N(%)	Mild TB N(%)	OR^a^(95% CI)	P^a^
GAG	202(18.1)	193(16.7)	1.100(0.885–1.367)	0.39
GGA	45(4.0)	32(2.8)	1.473(0.929–2.335)	0.10
GGG	189(17.0)	176(15.2)	1.134(0.906–1.419)	0.27
TAG	86(7.7)	88(7.6)	1.013(0.743–1.382)	0.93
TGA	510(45.7)	550(47.5)	0.923(0.782–1.089)	0.34
TGG	75(6.8)	106(9.2)	0.715(0.526–0.973)	0.032

Abbreviation: TB tuberculosis; SNPs single nucleotide polymorphisms; OR odds ratio; CI confidence interval; a, adjusted for age and gender

## Discussion

Cellular immunity plays a crucial role in the body’s immune system, and the pathogenesis of tuberculosis is closely associated with the immune function of the body. Cytokines, which are small molecular proteins secreted by cells, play essential mediatory and regulatory roles in various interactions between immune cells. The level of cytokines determines the immune function of the body [12]. The IL-12 family, comprising IL-12, IL-23, IL-27, and IL-35, plays a regulatory role in the immune system’s ability to combat infectious diseases, autoimmune diseases, and tumors [13]. IL-23, as a major member of the IL-12 family, is primarily produced by activated CD4 + T cells, dendritic cells, macrophages, B cells, and endothelial cells. It consists of two subunits, IL-23 P19 and IL-12 P40, corresponding to the receptors IL23R and IL-12Rβ1, respectively. Research indicates that IL-23 is a pivotal cytokine in the activation, proliferation, and differentiation of CD4 + T cells, and it can induce their proliferation. Additionally, IL-23 can enhance the activity of antigen-presenting cells such as dendritic cells and promote immune responses [14, 15]. IL-23 directly induces the secretion of interferon-γ (IFN-γ) and interleukin-12 (IL-12) by dendritic cells, suggesting that it activates antigen-presenting cells and enhances the activity of helper T cells (Th1). This activation, in turn, strengthens the phagocytic capabilities of macrophages, the cytotoxicity of natural killer cells, and the inflammatory response of tissues [16]. IL-12, a crucial factor in the Th1 cell immune response, promotes the secretion of IFN-γ by Th1 cells and NK cells. Since Mycobacterium tuberculosis is an intracellular bacterium residing within macrophages, IFN-γ effectively enhances macrophage activity against Mycobacterium tuberculosis. Some studies suggest that IL-12 production may be dependent on IL-23, indicating the significant role of IL-23 in the immune response to tuberculosis [17].

*IL23R* polymorphism is associated with a variety of diseases, such as autoimmune diseases, chronic diseases, and infectious diseases, among which *IL23R* polymorphism has been unequivocally established to exhibit associations with a plethora of ailments encompassing autoimmune disorders, chronic conditions, and infectious diseases [14–16]. Notably, the investigation of *IL23R* polymorphism primarily gained momentum within the realm of autoimmune diseases [18–23]. In the context of Crohn’s disease, a comprehensive analysis revealed that rs7517847 and rs11209026 exerted a protective effect, whereas rs10889677, rs1004819, and rs1495965 did not exhibit a statistically significant correlation with the aforementioned disorder [24]. Moreover, investigations have indicated that rs6682925 is linked to malignant neoplasms and coronary heart disease [21, 23]. Pertaining to infectious diseases, *IL23R* polymorphisms have been reported in association with viral infections, leprosy, and related manifestations. A genome-wide association study by Zhang identified rs3762318 as a protective factor for leprosy [25].

While the presence of *IL23R* gene polymorphisms has been associated with a wide array of diseases, its relationship with tuberculosis remains comparatively limited. Only three articles documented the association between *IL23R* gene polymorphisms and tuberculosis, focusing specifically on Chinese and Tunisian populations. Ben-Selma W conducted a study involving 150 healthy controls and 168 patients with pulmonary tuberculosis in Tunisia, elucidating the association of rs11209026 with susceptibility and severity of pulmonary tuberculosis [26]. In the Uygur population of China, Jiang DB identified the AA genotype at rs7518660 as a potential risk factor for TB (P < 0.0001, OR: 6.25, 95% CI: 3.85-10), while the GG genotype appeared to confere a protective effect against PTB (P < 0.0001, OR: 0.21, 95% CI: 0.13–0.32). Furthermore, the rs10889677 C allele and CC genotype were suggested to be potential risk factors for TB (P = 0.0446, OR: 1.53, 95% CI: 1.01–2.31), whereas the AA genotype exhibits a protective effect [27].Furthermore, in the Chinese Uygur population, a noteworthy association was established between rs1884444 and cavitary lesions (GG + GT vs. TT, OR = 3.61, 95% CI: 1.90–6.85). Additional investigations have revealed that IL23R gene polymorphisms are implicated in osteoarticular tuberculosis among the Guangxi Zhuang population. The presence of the C allele of rs10489629 may act as a susceptibility factor for osteoarticular tuberculosis (OR = 0.657, 95% CI: 0.446–0.969), while the A allele of rs10889675 may also confer susceptibility to osteoarticular tuberculosis (OR = 0.6248, 95% CI: 0.4154–0.9397). Moreover, the CA genotype may be associated with susceptibility to osteoarticular tuberculosis [28]. Based on the aforementioned studies, it is evident that IL23R gene polymorphisms are associated with tuberculosis susceptibility and severity; however, the number of investigations conducted in this field remains limited, with a lack of studies specifically focusing on the Chinese Han population. Considering the crucial role played by IL23 in the immune response against tuberculosis, we aimed to further explore the potential association between IL23R polymorphisms and the susceptibility as well as severity of tuberculosis. Within our study, we did not observe a significant association between the IL23R gene polymorphisms rs1884444, rs7518660, rs7539625 and tuberculosis. Notably, rs7518660 was previously implicated in tuberculosis susceptibility among the Uygur population in China. Discrepancies between our findings and those reported in the literature may be attributed to variations in ethnic backgrounds. Nonetheless, in the comparison between severe tuberculosis and mild tuberculosis, the G allele of rs1884444 and the additive genetic model demonstrated a significantly increased risk of severe tuberculosis after adjusting for age and sex (OR^a^: 1.199, 95% CI: 1.009–1.424; P^a^=0.039, respectively). Subgroup analysis, accounting for confounding factors such as age and sex, also indicated that the, rs1884444 G allele was associated with an elevated risk of severe pulmonary tuberculosis and tuberculous meningitis.

The rs1884444 is located on exon 2 of IL23R and the non-synonymous SNP results in amino acid change in codon 3 (His3Gln). Therefore, it may affect the specificity and affinity of the ligand receptor, regulating the pro-inflammatory effect of Th17 cells and influencing the host immune response to tuberculosis. Previous studies have shown that mutations in this site may interfere with binding of an exonic splicing enhancer leading to exon skipping, malformation or alternative splicing [29]. Consequently, our investigation unveils that IL23R gene polymorphisms do not appear to be associated with tuberculosis susceptibility in the Chinese Han population. However, it does indicate a potential association between IL23R gene polymorphism and an elevated risk of severe tuberculosis.

To the best of our current knowledge, limited studies have identified an association between IL23R gene polymorphisms and the severity of tuberculosis. One such association involves the rs11209026 polymorphism, which has been linked to the severity of active tuberculosis in the Tunisian population. Another association involves the rs1884444 polymorphism, which has shown a significant correlation with cavitary lesions in Chinese Uygurs. In our study, we categorized severe tuberculosis into three subgroups: severe PTB, multisystemic TB, and tuberculous meningitis. Within the Han Chinese population, we observed that the IL23R polymorphism rs1884444 was associated with an increased risk of severe tuberculosis and tuberculous meningitis. These findings align with previous research conducted on the Chinese Uyghur population [27]. However, we did not find an association between rs1884444 and the risk of multisystemic TB, which warrants further investigation. It is important to note that the severity of tuberculosis can be influenced by the strain of Mycobacterium tuberculosis. In our study, we mainly focused on tuberculosis patients from Sichuan Province, where the predominant strain is the Beijing type, accounting for approximately 76% [30]. Therefore, we made efforts to minimize the impact of Mycobacterium tuberculosis strains on the severity of tuberculosis as much as possible.

Despite advancements in TB diagnostic technology, the number of TB-related deaths remains high. According to the 2022 Global TB data report, it is estimated that China alone has 30,000 TB-related deaths, with a TB mortality rate of 2.1 per 100,000 population and a case fatality rate of 4% [1]. Severe TB stands as the primary cause of mortality, with the mortality rate for TB alone ranging from 22.4 to 68.2% between the 1970s and 2011 [31]. Given the high mortality rate associated with severe TB, the lack of standardized definitions, guidelines, and comprehensive understanding of the complex pathogenesis underlying severe TB, numerous genetic mechanisms remain unexplained. Hence, our aim was to delve deeper into the influence of host gene single nucleotide polymorphisms on severe TB. Through our study, we have identified a noteworthy association between IL23R gene polymorphisms and the risk of severe TB.

Our study has several limitations that should be acknowledged. Firstly, the lack of standardized criteria and guidelines for defining severe tuberculosis posed a challenge in our study. We relied on partial guidelines and clinical expertise to define severe tuberculosis, which may have introduced variability in the inclusion criteria and potentially influenced our results. Secondly, our investigation focused only on three specific tagSNP loci (rs1884444, rs7518660 and rs7539625), which may not represent the full spectrum of genetic variations in the IL23R gene. Thirdly, the susceptibility and severity of tuberculosis are influenced by multiple factors, including genetic, environmental, and gene-environment interactions. We did not account for important environmental factors such as smoking and alcohol consumption, which may have influenced the outcomes. Fourthly, our study was limited to the Chinese Han population aged between 15 and 45 years old, and further validation in other populations is necessary. It is crucial to exercise caution when generalizing these findings to other ethnic groups. Fifthly, our study focused solely on the impact of IL23R gene single nucleotide polymorphisms on tuberculosis susceptibility and severity. Further functional investigations are required to confirm our conclusions. Additionally, the statistical significance may be compromised by multiple comparisons adjustment considering the number of SNPs and subgroups analyzed. Future studies with larger sample sizes are warranted to address these limitations.

Despite these limitations, our research has made a significant contribution by revealing that IL23R gene polymorphism may not be associated with tuberculosis susceptibility in the Chinese Han population but may confer an increased risk of severe tuberculosis.

## Electronic supplementary material

Below is the link to the electronic supplementary material.

Supplementary Material 1

Supplementary Material 2

Supplementary Material 3

## Data Availability

The datasets utilized and/or analyzed during the current study are available from the corresponding author upon reasonable request.
