# Supplementary figures and images for: Association between a single nucleotide polymorphism of the IL23R gene and tuberculosis in a Chinese Han population: a case‒control study
Source: BMC Pulm Med. 2023 Jul 18;23:265. doi: 10.1186/s12890-023-02546-w (PMC10354923; doi:10.1186/s12890-023-02546-w)

Supplementary Figure 1 Linkage disequilibrium (LD) of all four tagSNPs displayed in the form of r2


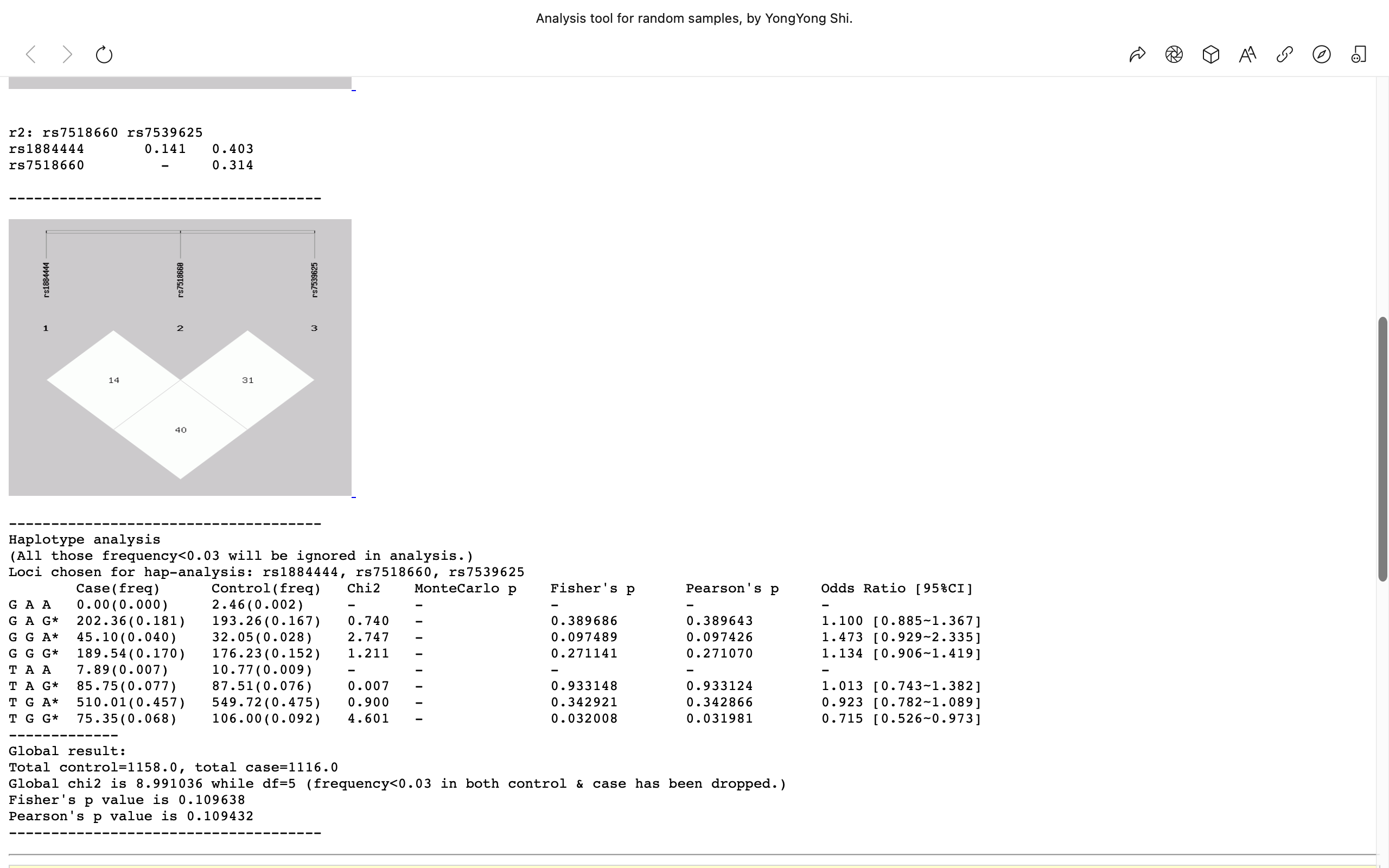

Supplement: Supplementary file 1 — Supplementary Material 1 [file 12890_2023_2546_MOESM1_ESM.docx]
