# Supplementary material for: Association between a single nucleotide polymorphism of the IL23R gene and tuberculosis in a Chinese Han population: a case‒control study
Source: BMC Pulm Med. 2023 Jul 18;23:265. doi: 10.1186/s12890-023-02546-w (PMC10354923; doi:10.1186/s12890-023-02546-w)
Supplement: Supplementary file 2 — Supplementary Material 2 [file 12890_2023_2546_MOESM2_ESM.docx]

Supplementary Table 1 Characteristics of TB patients and healthy controls

| Features | TB patients N(%） | | | | total | Control group N(%） | P# |
| --- | --- | --- | --- | --- | --- | --- | --- |
|  | Severe TB | | | Mild TB |  |  |  |
|  | Severe PTB | multisystemic tuberculosis TB | Tubercular meningitis |  |  |  |  |
| Number | 219 | 286 | 53 |  |  |  |  |
| Total | 558 | | | 579 | 1137 | 581 |  |
| Mean Age | 27.92±8.254 | | |  |  | 27.97±6.093 | 0.899 |
| Males | 606（53.3） | | |  |  | 302（52） | 0.604 |
| Females | 531（46.7） | | |  |  | 279（48） |  |

Abbreviation: TB Tuberculosis,PTB pulmonary tuberculosis
