# Supplementary material for: Association between a single nucleotide polymorphism of the IL23R gene and tuberculosis in a Chinese Han population: a case‒control study
Source: BMC Pulm Med. 2023 Jul 18;23:265. doi: 10.1186/s12890-023-02546-w (PMC10354923; doi:10.1186/s12890-023-02546-w)
Supplement: Supplementary file 3 — Supplementary Material 3 [file 12890_2023_2546_MOESM3_ESM.docx]

Supplementary Table 2 Frequency distribution of IL23R gene polymorphisms in TB patients and healthy controls.

| SNPs | Allele/  Genotype | Severe PTB group  vs Mild TB group | |  | Multisystem TB group vs Mild TB group | |  | tubercular meningitis group vs Mild TB group | |  | Mild TB group vs Healthy control group | |  | Severe TB group vs Healthy control group | |
| --- | --- | --- | --- | --- | --- | --- | --- | --- | --- | --- | --- | --- | --- | --- | --- |
|  |  | Severe PTB group N(%) | Mild TB group  N(%) |  | Multisystem TB group  N(%) | Mild TB group  N(%) |  | tubercular meningitis group N(%) | Mild TB group  N(%) |  | Mild TB group  N(%) | Healthy control group  N(%) |  | Severe TB group  N(%) | Healthy control group  N(%) |
| rs18844444（T>G） | T | 258（58.9） | 754（65.1） |  | 365(63.8) | 754(65.1) |  | 56（52.8） | 754（65.1） |  | 754（65.1） | 731（62.9） |  | 679(60.8) | 731(62.9) |
|  | G | 180（41.1） | 404（34.9） |  | 207(36.2) | 404(34.9) |  | 50（47.2） | 404（34.9） |  | 404（34.9） | 431（37.1） |  | 437(39.2) | 431(37.1) |
|  | TT | 82（37.4） | 250（43.2） |  | 114(39.9) | 250(43.2) |  | 15（28.3） | 250（43.2） |  | 250（43.2） | 234（40.3） |  | 211(37.8) | 234(40.3) |
|  | GT | 94（42.9） | 254（43.9） |  | 137(47.9) | 254(43.9) |  | 26（49.1） | 254（43.9） |  | 254（43.9） | 263（45.3） |  | 257(46.1) | 263(45.3) |
|  | GG | 43（19.6） | 75（13.0） |  | 35(12.2) | 75(13.0) |  | 12（22.6） | 75（13.0） |  | 75（13.0） | 84（14.5） |  | 90(16.1) | 84(14.5) |
| Rs7518660（G>A） | G | 314(71.7) | 864(74.6) |  | 432(75.5) | 864(74.6) |  | 74(69.8) | 864(74.6) |  | 864(74.6) | 859(73.9) |  | 820(73.5) | 859(73.9) |
|  | A | 124(28.3) | 294(25.4) |  | 140(24.5) | 294(25.4) |  | 32(30.2) | 294(25.4) |  | 294(25.4) | 303(26.1) |  | 296(26.5) | 303(26.1) |
|  | GG | 114(52.1) | 322(55.6) |  | 163(57.0) | 322(55.6) |  | 25(47.2) | 322(55.6) |  | 322(55.6) | 318(54.7) |  | 302(54.1) | 318(54.7) |
|  | GA | 86(39.3) | 220(38.0) |  | 106(37.1) | 220(38.0) |  | 24(45.3) | 220(38.0) |  | 220(38.0) | 223(38.4) |  | 216(38.7) | 223(38.4) |
|  | AA | 19(8.7) | 37(6.4) |  | 17(5.9) | 37(6.4) |  | 4(7.5) | 37(6.4) |  | 37(6.4) | 40(6.9) |  | 40(7.2) | 40(6.9) |
| Rs7539625（A>G） | A | 227(51.8) | 563(48.6) |  | 287(50.2) | 610(52.7) |  | 50（47.2） | 595（51.4） |  | 595(51.4) | 621(53.4) |  | 563(50.4) | 621(53.40 |
|  | G | 211(48.2) | 595(51.4) |  | 285(49.8) | 548(47.3) |  | 56（52.8） | 563（48.6） |  | 563(48.6) | 541(46.6) |  | 553(49.6) | 541(46.6) |
|  | AA | 64(29.2) | 144(24.9) |  | 81(28.3) | 160(27.6) |  | 9（17.0） | 160（27.6） |  | 160(27.6) | 170(29.3) |  | 146(26.2) | 170(29.3) |
|  | GA | 99(45.2) | 275(47.5) |  | 140(49.0) | 275(47.5) |  | 32（60.4） | 275（47.5） |  | 275(47.5) | 281(48.4) |  | 271(48.6) | 281(48.4) |
|  | GG | 56(25.6) | 160(27.6) |  | 65(22.7) | 144(24.9) |  | 12（22.6） | 144（24.9） |  | 144(24.9) | 130(22.4) |  | 141(25.3) | 130(22.4) |

Abbreviation: TB tuberculosis;SNPs single nucleotide polymorphisms; OR odds ratio; CI confidence interval; a,adjusted for age and gender
